# Resistance to Antibiotics and Antifungal Medicinal Products: Can Complementary and Alternative Medicine Help Solve the Problem in Common Infection Diseases? The Introduction of a Dutch Research Consortium

**DOI:** 10.1155/2015/521584

**Published:** 2015-10-11

**Authors:** Esther T. Kok, Miek C. Jong, Barbara Gravendeel, Willem B. Van Leeuwen, Erik W. Baars

**Affiliations:** ^1^University of Applied Sciences, 2333 CK Leiden, Netherlands; ^2^Centre for Academic Primary Care, School of Social and Community Medicine, University of Bristol, Bristol BS8 2PS, UK; ^3^Department of Healthcare and Nutrition, Louis Bolk Institute, 2972 LA Driebergen, Netherlands; ^4^Department of Health Sciences, Mid Sweden University, 871 31 Sundsvall, Sweden; ^5^National Information and Knowledge Centre on Integrative Medicine (NIKIM), Amsterdam, Netherlands; ^6^Naturalis Biodiversity Center, 2333 CR Leiden, Netherlands

## Abstract

The increase of antibiotic resistance worldwide, rising numbers of deaths and costs associated with this, and the fact that hardly any new antimicrobial drugs have been developed during the last decade have increased the interest in Complementary and Alternative Medicine (CAM) therapeutic interventions, if proven safe and effective. Observational studies on clinical CAM practices demonstrate positive effects of treatment of infections with CAM therapies (clinical effects, patient satisfaction) in combination with small percentages of antibiotics prescription. However, Cochrane reviews and other studies demonstrate that in most instances the quality of clinical trials on CAM treatment of infections is currently too low to provide sufficient evidence. Therefore a Dutch consortium on (in vitro and clinical) scientific research on CAM and antibiotic resistance has been formed. The aim and objective of the consortium is to establish an enduring partnership and to develop expertise to further develop and investigate safe and effective CAM treatments for infectious diseases of humans (and animals). A first ongoing project on the development of safe and effective biobased CAM antimycotics in women with (recurrent) vaginal candidiasis infection is introduced.

## 1. Introduction

The discovery of antibiotics was one of the most significant events in medical history and is said to have added a decade to the life expectancy of humans [1]. Together with vaccination and public health measures (e.g., clean water, invention, and introduction of drainage systems and the fridge), antibiotics were responsible for a dramatic reduction of the mortality rate from infectious diseases. Regrettably, it seems that the successful use of any therapeutic agent is compromised by the potential development of tolerance or resistance to that compound from the time it is first employed.

The development of generations of antibiotic-resistant microbes and their distribution are the result of many years of underuse, overuse, and misuse of antibiotics by human applications (weak or no antibiotic policy and poor infection control). For more than five decades the problem of how to contain antimicrobial resistance (AMR) persists and due to the low chance of success we are now, in the early decades of the 21st century, facing a global issue of concern with serious consequences: “Drug-resistant infections already kill hundreds of thousands a year globally, and by 2050 that figure could be more than 10 million. The economic cost will also be significant, with the world economy being hit by up to $100 trillion by 2050 if we do not take action [2].” Therefore, worldwide actions are established to disprove the hypothesis of Felix Marti-Ibanezin, already stated in 1955: “Antibiotic therapy, if indiscriminately used, may turn out to be a medicinal flood that temporarily cleans and heals, but ultimately destroy life itself” [3].

The fact that hardly any new antimicrobial drugs have been developed during the last decade has increased the interest in Complementary and Alternative Medicine (CAM) therapeutic interventions, if proven safe and effective. CAM interventions can contribute to a reduction in antibiotic use by (1) strengthening the self-healing capacities of the individual and/or (2) providing an alternative treatment which has its own antimicrobial effect [4, 5].

In this paper the introduction of CAM in the future control of AMR and examples of the evidence on CAM alternatives are presented. A Dutch consortium of research partners is initiated to (further) develop safe and effective CAM alternatives to antibiotics.

## 2. Future Control of AMR

The environmental and policy factors that contribute to resistance require regulatory and governmental intervention. Therefore actions for future control are being taken to determine (research) and promote (education) appropriate use of antimicrobial drugs on national and international basis.

Ongoing research is being performed to obtain evidence for sophisticated molecular, immunologic, and microbial techniques that will change the way infectious diseases are diagnosed in the hope to reduce diagnostic uncertainty in the next 2 decades [6]. Furthermore the development of new vaccines might contribute to a decreased transmission and impact of antimicrobial-resistant bacteria in the near future as these vaccines might have the potential to effectively control infectious agents [7]. However, vaccine escape mechanisms for bacteria have already been reported [8]. Variability in parts of the genome coding for antigenic determinants, such as in* Bordetella pertussis* (whooping cough), may lead to a vaccine escape. In this way, despite vaccination, this DNA variability contributed to reemergence of whooping cough in the Netherlands.

National and international antimicrobial drug policies and guidelines on infection control have been developed and implemented resulting in a decrease of the volume of antimicrobials over the past 10 years [9, 10]. Besides, national campaigns to educate physicians and patients about the appropriate use of antibiotics have been launched resulting in promising changes in attitude among the public and healthcare professionals [11].

Whether the current epidemic of AMR is sustainable or will succumb to the current efforts will also depend on the worldwide healthcare regulation, as antimicrobial use is affected by reimbursement policies, financial incentives, and healthcare regulation [12].

However, even if the use of antibiotics was entirely appropriate resistance would still occur. Hence, the development of new pharmaceuticals and antimicrobial agents is essential and recommendations to facilitate development of new antimicrobial agents are listed [13, 14]. One of these recommendations is to increase the cooperation between academia and industry for identifying potential targets. Potential targets might be vaccination, surgical interventions, awareness, and control of environmental risk factors, as well as complementary and alternative medicine (CAM) [15].

## 3. Complementary and Alternative Medicine

Consumer interest in CAM has increased over the past decade. Recent data indicate that a large proportion of the population of developed countries, including Australia (52–69% of those surveyed), Canada (59-60%), the United States (62%), Singapore (76%), and Japan (50%), has used CAM at least once over a twelve-month period [16]. Between 20% and 80% of citizens in different EU countries have used CAM in their healthcare [17]. In the Netherlands 15 percent of the total population visits a CAM therapist yearly [18].

There are several explanations for the increasing use of CAM services across the world. Earlier studies have suggested that consumer dissatisfaction with conventional medication may be a leading reason for CAM use [16]; however, more recent reports indicate that an aspiration for active healthcare participation, greater disease chronicity and severity, holistic healthcare beliefs, and increase in health awareness behaviour are more likely to be associated with CAM use [19–21]. This suggests that complementary medicine is addressing unmet needs in healthcare.

Due to the worldwide increasing use of CAM services, the attention for safety, effectiveness, and cost-effectiveness of CAM grows. A previous review [22] reported that some CAM therapies are cost-effective compared with usual care for various conditions. More recent results of two Dutch studies demonstrated that patients whose general practitioner (GP) practices CAM tend to have lower costs (10.1%) [23, 24].

The effectiveness of many CAM therapies has not been proven in clinical trials yet [25]. Randomized controlled trials (RCTs) are still considered and applied as the golden standard for evaluation of effectiveness. However, RCTs may not always be suitable for the evaluation of CAM, in particular, to investigate the individual response treatment. The description of individual cases is probably the most important tool for teaching in medicine, especially in CAM. However, most of the times the information provided in single case reports is not sufficient to provide sufficient evidence for effectiveness [26]. Development of other methods for evaluation of individual response instead of RCTs or adapted in RCTs is necessary as it is essential that scientific evidence for CAM will be established. In the best case, once scientific evidence is there to prove a CAM therapy safe and effective and when there is a clear working mechanism, the status can change from CAM into conventional medicine.

Between January 2010 and December 2012, the CAMbrella consortium has been looking into the current status of CAM in Europe from different angles. Their findings stated that future research methods must reflect the real-world settings of healthcare in Europe and that everyone needs to know in what situation CAM is a reasonable choice [27]. Therefore they recommend a clear emphasis on concurrent evaluation of CAM as an additional or alternative treatment strategy in real-world settings. The strategy for the investigation of CAM should include a broad range of mixed-method research strategies including comparative effectiveness research and qualitative and quantitative designs. Stakeholders such as citizens, patients, and providers should be closely involved to ensure real-world relevance for the research [27].

## 4. CAM Alternatives to Antibiotics

CAM can contribute to a reduction in antibiotic use. On the one hand, CAM therapies, which are proven safe, can be used to strengthen the self-healing capacities of the organism (preventive and curative health promotion) [4]. Here CAM is an alternative for antibiotics but is not directly based on the antimicrobial properties of the product itself. For example, compared to conventional treatment, anthroposophic treatment of primary care patients with acute respiratory and ear symptoms had more favourable outcomes, lower antibiotic prescription rates, less adverse drug reactions, and higher patient satisfaction [28]. Moreover, the introduction of CAM medicinal products might overcome the related side effects of antibiotic use in childhood as several studies show that both maternal and child's use of antibiotics were associated with an increased risk of eczema or asthma [29–31].

On the other hand, several (natural) medicinal products as used in CAM can act as an alternative (fighting disease strategies) to control infectious diseases based on their own (bactericide or bacteriostatic) antimicrobial properties [5].

The development of conventional medicinal products and CAM medicinal products such as those used in anthroposophic medicine, homeopathy, and traditional Chinese medicine follow different pathways. Conventional medicinal products are developed in the laboratory and subsequently tested in preclinical studies, phases 1–3 clinical studies, and finally in clinical practice. They are mostly used according to a fixed schedule and for one indication. Conversely, CAM medicinal products are developed in clinical practice with regard to the principles of the respective medical system, mostly have a long tradition of use, are often selected individually to address the needs of each patient on the basis of trained judgment skills of the health practitioner, and are often used in combination with other (conventional) medicinal products or nonpharmacological therapies. As a result, CAM usually has multiple therapy options for each indication [32].

Overall, there is much expert knowledge on CAM treatment of infectious diseases but little scientific evidence based on clinical trials. Currently there are 61 Cochrane reviews on CAM treatments of specific infections (e.g., 29 on respiratory tract infections). However, in most instances the quality of clinical trials on CAM treatment of infections reviewed is currently too low to provide sufficient evidence. Nevertheless, some reviews and observational studies do demonstrate positive and promising results.

For example, an observational study on anthroposophic clinical practice demonstrates positive effects of treatment of acute middle ear and upper respiratory infections (clinical effects, patient satisfaction) in combination with small percentages of antibiotics prescription [28].

With regard to positive reviews,* Echinacea* is one of the most widely used botanical supplements in North America in the treatment of upper respiratory tract infections. One review of 34 studies using* Echinacea* for the prevention of upper respiratory tract infections showed that 22 had positive outcomes [33]. Another review of nine RCTs showed that 8 reported some benefit of* Echinacea* in the early treatment of upper respiratory infections [34].

The good antimicrobial properties of* Abrus precatorius*,* Terminalia phanerophlebia*,* Indigofera arrecta*, and* Pentanisia prunelloides* authenticate their traditional use in treatment of respiratory diseases [35].

The increasing prevalence of isolates of* Escherichia coli* (the most prevalent uropathogen) that are resistant to antimicrobial agents has stimulated interest in novel nonantibiotic methods for prevention of urinary tract infections (UTIs). Cranberries have been used in the prevention of UTIs for many years. A meta-analysis of the results of two well-performed RCTs showed that, in women with recurrent UTIs, cranberry products reduced the incidence of recurrences at 12 months by 39% compared with placebo or control interventions [36]. Beerepoot et al. reported that in premenopausal women the use of antibiotics (TMP-SMX) is more effective than cranberry capsules to prevent recurrent UTIs. However, the use of TMP-SMX resulted in a considerable increase in antibiotic resistance [37].

Another example is found in tackling antibiotic-resistant strains of pathogens as* Candida albicans*. Candidiasis is a benign mycosis resulting from a yeast infection caused by* Candida albicans*.* Candida albicans* develops resistance to regularly applied, standard antimycotic drugs such as clotrimazole, nystatin, fluconazole, and ketoconazole [38], and, as a consequence, demand for prevention is high. Worldwide several plant extracts have traditionally been used to prevent Candidiasis [39]. The drawback with these treatments is that they might interact with other medications if taken together through changes in drug solubility and uptake, metabolism, and physiology of the gastrointestinal tract [40]. This results in adverse drug reactions such as allergic reactions, reduced effects of contraceptives, and stomach damage [41]. Therefore research to profile, detect, and screen alternatives among, for example, plant compounds, which are effective in preventing Candidiasis without these shortcomings, is needed.

## 5. Scientific Evidence as Key

Worldwide research in CAM is seriously hampered by a lack of research infrastructure and funding, lack of research expertise among CAM practitioners, lack of appropriate research models and strategies, and the scepticism of the conventional scientific community. In the USA, the national authorities have taken the growing demand for CAM seriously. The established National Centre for Complementary and Alternative Medicine (NCCAM) in 1998 has already funded 57 university based centres for research on CAM, in contrast to Europe where only some western countries (Denmark, Germany, Norway, the UK, and the Netherlands) have granted some money for research projects in CAM [42].

CAM research might contribute to controlling AMR and in broader context to improving health, reducing disease, and reducing healthcare related costs. However, the CAM industry alone cannot be expected to support all research activities in these areas. At the moment there is huge disparity between public funding for conventional drug research and that for CAM research.

Nowadays, national health authorities are asking for effective actions to control AMR, and therefore the need for sound clinical research (methods) to test the efficacy for CAM strategies becomes more important. Clinicians using CAM in daily routine practice, often resulting in nonuse or extended use of antibiotics, are convinced about the effectiveness of their services. However, before general acceptation and introduction of these specific CAM alternatives first scientific evidence is needed. Clearly, scientific evidence of CAM based on sound clinical research methodology is the key to action.

## 6. The Introduction of a Consortium

The Professorships of Anthroposophic Healthcare, Biodiversity, and Innovative Molecular Diagnostics of the University of Applied Services of Leiden and the Louis Bolk Institute in the Netherlands have taken the initiative to form a consortium on scientific research on CAM and antibiotic resistance (see Appendix). The aim and objective of the consortium is to establish an enduring partnership and to develop expertise to further develop and investigate safe and effective CAM treatments for infectious diseases of humans (and animals). The knowledge generated will lead to (1) evidence-based CAM alternatives to antibiotics that can be used in clinical practice and (2) guidelines for CAM treatments for infectious diseases in human and veterinary clinical practice.

## 7. Description of First Project

A first project to find close relatives of species traditionally used to prevent Candidiasis that could serve as safe biobased antimycotics to be applied in registered medical sprays has started (granted by the Naturalis tender application-oriented research 2013). In short the study exists out of the following activities:

1. Instead of using random screening, a multistep targeted approach is used that incorporates information retrieved from museum collection labels to optimize efficient species selection.
  - Species across the angiosperms that have uncomplicated European Medicines Agency (EMA) regulations will be selected.
  - Phylogenetic prospecting will be applied to identify closely related species of interest.
  - Crucial information of commercial interest, that is, cultivation requirements and traditional use, will be retrieved from metadata attached to specimens, screened for legal constraints, and applied in the prospecting.
2. Extracts of living accessions from the profiled species are being tested in antifungal and cellular assays.
3. The most promising extract will be added to a registered medical device (using good manufacturing practice guidelines) and will after ethics approval be tested on safety and efficacy using a double blind-placebo controlled trial design in clinical practice.

## 8. Conclusion

The increasing incidence of drug-resistant pathogens has drawn the attention of the pharmaceutical and scientific communities towards studies on the potential antimicrobial activity of CAM products and therapies. The aim of the introduced Dutch CAM consortium is to provide evidence of safety, efficacy, cost (effects), and modes of actions of CAM therapies, which are useful as alternative strategies to control infectious diseases and can become useful therapeutic tools in clinical practice. The Dutch consortium will serve as a starting point for further international collaboration with stakeholders involved and/or interested in the study of CAM contributions to the treatment of infections and the reduction of AMR.

## Appendix

### Composition of the Consortium

The consortium consists of the following parties:

1. *University of Applied Sciences Leiden (Hogeschool Leiden)* participates with three professorships as follows:
  - *Anthroposophic Healthcare* (Professor Dr. E. W. Baars and Dr. E. T. Kok): it is able to cooperate with the Louis Bolk Institute to perform clinical studies to the safety and effects of (new) CAM treatments in patients with infectious diseases. The research group also has a large network with anthroposophic care professionals including doctors and nurses.
  - *Biodiversity* (Professor Dr. B. Gravendeel) and* Innovative Molecular Diagnostics* (Professor Dr. W. van Leeuwen): both research groups are able to investigate the in vitro effects and safety of (new) CAM treatments for infectious diseases.
2. *Louis Bolk Institute* (Dr. M. C. Jong): it is able to collaborate with the professorship of Anthroposophic Healthcare to perform clinical studies to the safety and effects of CAM treatments in patients with infectious diseases. The institute also has a large network with CAM practitioners in the Netherlands.

## Abbreviations

AHI: Animal Health Institute
AMR: Antimicrobial resistance
CAHCIM: Consortium of Academic Health Centres for Integrative Medicine
CAM: Complementary and Alternative Medicine
CDC: Centres for Disease Control and Prevention
EMA: European Medicines Agency
FDA: Food and Drug Administration
GP: General practitioner
IM: Integrative medicine
NCCAM: National Center for Complementary and Alternative Medicine
WHO: World Health Organization.
